# Influence of arthritis-related protein (BBF01) on infectivity of *Borrelia burgdorferi* B31

**DOI:** 10.1186/1471-2180-13-100

**Published:** 2013-05-07

**Authors:** Denise Imai, Kevin Holden, Eric M Velazquez, Sunlian Feng, Emir Hodzic, Stephen W Barthold

**Affiliations:** 1 Center for Comparative Medicine, Schools of Veterinary Medicine and Medicine, University of California, One Shields Avenue, Davis, CA 95616, USA

**Keywords:** Lyme borreliosis, Arthritis related protein, BBF01

## Abstract

**Background:**

Lyme borreliosis, caused by tick-borne *Borrelia burgdorferi*, is a multi-phasic, multi-system disease in humans. Similar to humans, C3H mice develop arthritis and carditis, with resolution and periodic bouts of recurrence over the course of persistent infection. *Borrelia burgdorferi* arthritis-related protein (Arp/BBF01), a highly conserved protein among *B. burgdorferi* s.s. isolates, has been shown to be antigenic in humans with Lyme borreliosis, and a target for antibody-mediated disease resolution in the mouse model.

**Results:**

A mutant strain of *B. burgdorferi* s.s. deficient of the *arp* gene and a complemented version of that mutant were created and examined for phenotypic effects in mice compared to wild-type *B. burgdorferi*. Deletion of *arp* did not abolish infectivity, but did result in a higher infectious dose compared to wild-type *B. burgdorferi*, which was restored by complementation. Spirochete burdens in tissues of C3H-*scid* mice were lower when infected with the *arp* mutant, compared to wild-type, but arthritis was equally severe. Spirochete burdens were also lower in C3H mice infected with the *arp* mutant, but disease was markedly reduced. Ticks that fed upon infected C3H mice were able to acquire infection with both wild-type and *arp* mutant spirochetes. Arp mutant spirochetes were marginally able to be transmitted to naïve hosts by infected ticks.

**Conclusion:**

These results indicated that deletion of BBF01/*arp* did not abrogate, but diminished infectivity and limited spirochete burdens in tissues of both immunocompetent and immunodeficient hosts, and attenuated, but did not abolish the ability of ticks to acquire or transmit infection.

## Background

Lyme disease, caused by tick-borne *Borrelia burgdorferi,* is a multi-systemic and multi-phasic disease in humans, which includes pauciarticular arthritis in up to 60% of untreated patients [1,2]. In the absence of antibiotic treatment, arthritis and other lesions undergo resolution with variable bouts of recurrence over the course of months to years of persistent infection [3]. Laboratory mice develop arthritis and carditis that follow a similar multi-phasic course as humans, with resolution and periodic bouts of recurrence over the course of persistent infection [4]. The mouse model has implicated the humoral immune response as a critical factor in arthritis and carditis resolution. Infection of T-cell deficient (*Tcr* α/βnull, *Tcr* γ/δ-null), but not B-cell deficient (*Igh6*-null) or severe combined immunodeficient (SCID) or *Rag1*-null mice follows a course of resolution that is similar to fully immunocompetent mice [5], and passive transfer of serum from actively infected immunocompetent mice that have undergone disease resolution (immune serum) into infected SCID mice results in complete resolution of arthritis and carditis, but not clearance of infection [6-8].

Identification of the *B. burgdorferi* antigens targeted by antibodies that mediate disease resolution is complicated by the fact that *B. burgdorferi* grown in culture medium does not reflect the antigenic profile of spirochetes during mammalian infection [9,10]. As a means to identify vulnerable antigenic targets that are expressed in the mammalian host that are responsible for antibody-mediated disease resolution, immune serum from actively infected mice has been used to probe *B. burgdorferi* genomic expression libraries or outer membrane extracts. These efforts revealed arthritis-related protein (BBF01/Arp) as well as decorin binding protein A (DbpA), among other antigens expressed during infection [8,11-13]. Antiserum generated in mice hyperimmunized with non-lipidated recombinant Arp or DbpA induced arthritis and carditis resolution, but did not eliminate infection, when passively transferred to actively infected SCID mice [8,12]. Immunization with DbpA was found to induce protective immunity against cultured spirochetes [11,14], but not tick-borne spirochetes [15], whereas Arp immunization was ineffective at eliciting protective immunity against cultured spirochetes [16]. Outer surface protein C (OspC), another immunogenic protein expressed during infection, has also been shown to be vulnerable to passively transferred OspC antibody in SCID mice, but is down-regulated in response to specific antibody, thereby avoiding immune clearance in immunocompetent mice [17,18].

Based upon the presence of a leader sequence, *arp* encodes a putative lipoprotein that is expressed in ticks and throughout the course of mammalian infection and has been shown to stimulate the production of antibody in both experimentally infected mice and naturally infected humans [13,16,19-21]. Genomic comparison among several *B. burgdorferi* sensu stricto (s.s.) strains reveals highly conserved BBF01/*arp* sequences (95-100% identity from GenBank Blast). Curiously, the genomes of other *B. burgdorferi* sensu lato strains that are available in GenBank, such as *B. afzelii* and *B. garinii*, do not appear to have an *arp* homolog. In contrast to *arp* conservation in *B. burgdorferi* s.s. strains, *dbpA* and *ospC*, which also encode immunogenic antigens that are expressed during infection [19,21-23], have considerable variation (81-85% identity) among the same *B. burgdorferi* s.s. strains (GenBank). As noted, both Arp and DbpA stimulate an arthritis-resolving immune response [8], and DbpA and OspC elicit protective immune responses against challenge [11,14,24]. It is therefore curious that Arp has such a conserved sequence among *B. burgdorferi* s.s. strains, when it is so obviously subjected to immune selection pressure. The present study explored the biological behavior of *B. burgdorferi* devoid of, or complemented with, Arp. Arp was found to be non-essential for infectivity, but it influenced infectious dose, spirochete burdens in tissues, arthritis severity, and tick infection kinetics, underscoring its biological significance.

## Results

Seven *B. burgdorferi* B31-*arp* deletion mutants (Δarp) were created, and found to grow equally well in BSKII medium as B31 (wild-type) spirochetes. The 7 Δarp mutants were initially tested for infectivity in infant ICR mice, which serve as an inexpensive system for titrating infectivity [5]. All seven mutants were determined to be *flagellin B* (*flaB)* DNA-positive and *arp* DNA-negative by polymerase chain reaction (PCR), following growth selection in streptomycin. Four 2-day-old mice were inoculated with 10^6^ of each Δarp mutant or wild-type spirochetes, and sub-inoculation site and urinary bladder were cultured to determine infectivity and ability to disseminate at 7 and 21 days after inoculation. All were infectious, and all disseminated to the urinary bladder. Spirochetes cultured from the inoculation site and urinary bladder were tested by PCR for presence of *flaB* and *arp*. Urinary bladder isolates from mice that were *flaB*-positive and *arp*-negative were selected for further analysis and confirmed to be *arp*-null. Upon subsequent inoculation of infant ICR mice with wild-type or each of the seven Δarp mutants, arthritis was of equivalent severity as mice infected with B31 among all groups of mice, indicating that *B. burgdorferi* devoid of *arp* were not only infectious, but also equally pathogenic as wild-type *B. burgdorferi* in susceptible infant mice. One *arp* isolate (Δarp3) was selected for further analysis.

The median infectious dose (ID_50_) of Δarp3 was compared to wild-type and to Δarp3 complemented with the plasmid lp28-1G containing *arp* (Δarp3 + lp28-1G). Groups of 4 infant ICR mice were inoculated subdermally with 10^1^, 10^2^, 10^3^, 10^4^, or 10^5^ spirochetes. Mice were necropsied at 2 weeks, and sub-inoculation site and urinary bladder were cultured (Table 1). The ID_50_ of wild-type was 5×10^3^ spirochetes, whereas the ID_50_ of Δarp3 was 8×10^4^ spirochetes. Relative infectivity could be restored by complementation of the Δarp3 mutant with lp28-1G, resulting in an ID_50_ identical to wild-type. Subsequent experiments in C3H and C3H-*scid* mice therefore used an infectious dose of 10^5^ or greater spirochetes.

**Table 1:** **Dose-related infectivity of *arp *null (Δarp3), Δarp3-complemented (Δarp3 + lp28-1G) and wild-type *B. burgdorferi *in infant ICR mice, based upon culture of sub-inoculation site and urinary bladder at 2 weeks after inoculation**

**Inoculum dose**	**Δarp3**	**Δarp3 + lp28-1G**	**wild-type**
10^1^	0/4*	0/4	0/4
10^2^	0/4	0/4	0/4
10^3^	0/4	0/4	0/4
10^4^	1/4	4/4	4/4
10^5^	2/4	4/4	4/4

* number of positive mice/number of mice tested.

**Figure 1:**
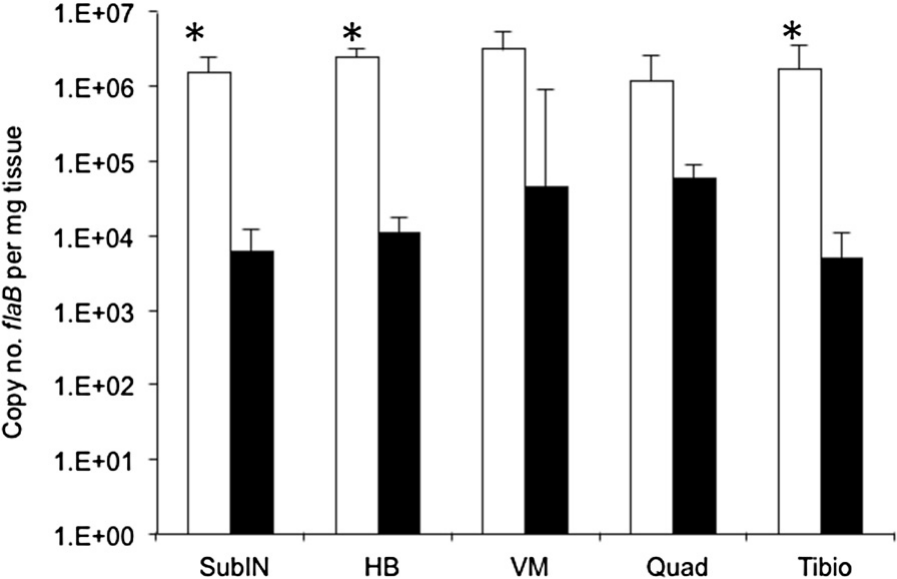
***Borrelia burgdorferi flaB *DNA copies per mg tissue weight (means ± standard deviations) in subinoculation site (subIN), heart base (HB), ventricular muscle (VM), quadriceps muscle (Quad) and tibiotarsus (Tibio) from 4 C3H-*scid *mice inoculated with wild-type (white bars) compared to 5 C3H-*scid *mice inoculated with *arp *null Δarp3 *B. burgdorferi *(black bars).** (*, *P *≤ 0.05).

Four C3H-*scid* mice were each inoculated with 10^6^ wild-type and five C3H-*scid* mice were each inoculated with 10^6^ Δarp3 spirochetes, and then necropsied at 60 days of infection to compare the full range of pathogenicity of each inoculum, unencumbered by acquired immunity. All inoculation sites and urinary bladders were culture-positive in both groups. Spirochetes were isolated from blood of 4/4 wild-type inoculated mice, whereas only 2/4 (one sample not collected) Δarp3 inoculated mice were bacteremic. All mice in both groups had severe (mean arthritis score 3.0 ± 0 SD) arthritis in tibiotarsal joints, as well as arthritis in both knees, and all mice had carditis. Despite equally severe disease, spirochete burdens in sub-inoculation, heart base, and tibiotarsal tissues, based upon *flaB* quantitative PCR (Q-PCR), were significantly lower (P ≤ 0.05) in Δarp3 infected C3H-*scid* mice compared to wild-type infected mice (Figure 1). Spirochete burdens were also lower in ventricular muscle and quadriceps muscle, but differences were not statistically significant.

A confirmatory experiment was performed in which 5 C3H-*scid* mice were each inoculated with 10^6^ wild-type and 5 C3H-*scid* mice were each inoculated with 10^6^ Δarp3 spirochetes, and necropsied on day 28 after inoculation. Inoculation sites and urinary bladders in all mice from both groups were culture-positive, and all mice in both groups were bacteremic. Arthritis severity scores were equivalent in both groups (mean 2.8 ± 0.4 SD wild-type vs. mean 2.4 ± 0.5 SD Δarp3). Significantly lower *flaB* Q-PCR spirochete burdens (*P* ≤ 0.05) were found in tissues of Δarp3 infected C3H-*scid* mice compared to wild-type infected C3H-*scid* mice, including inoculation site (320 ± 486 SD vs 249,660 ± 187,097 SD), heart base (983 ± 1,353 SD vs 514,400 ± 171,404 SD), ventricular muscle (9,656 ± 5,911 SD vs 432,260 ± 374,173 SD), and tibiotarsus (201 ± 187 SD vs 163,520 ± 89,127 SD). Spirochete burdens were also reduced in quadriceps muscle (3,730 ± 1,412 SD vs 58,640 ± 74,839 SD), but differences were not statistically significant (*P* = 0.07).

**Figure 2:**
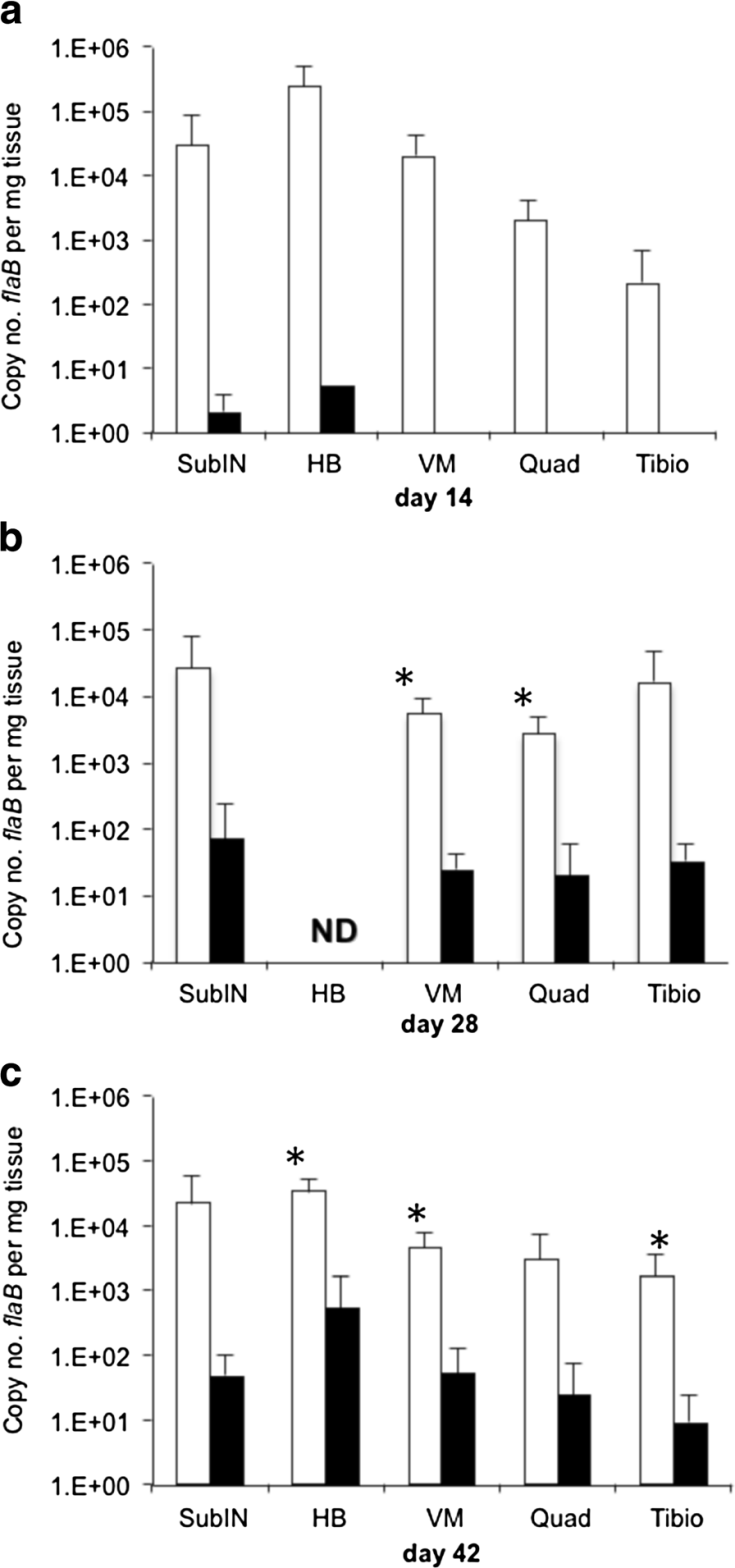
***Borrelia burgdorferi flaB *DNA copies per mg tissue weight (means ± standard deviations) in PCR-positive tissues summarized in Tables **2** and **3, including sub-inoculation site (subIN), heart base (HB), ventricular muscle (VM), quadriceps muscle (Quad) and tibiotarsus (Tibio) from C3H mice inoculated with wild-type (white bars) compared to *arp *null Δarp3 *B. burgdoferi *(black bars) at day 14 (a), day 28 (b) and day 42 (c) of infection. (*, *P *≤ 0.05) ND: not determined.

Next, groups of 5 immunocompetent C3H mice were inoculated with 10^5^ wild-type or Δarp3 spirochetes, and then necropsied on days 14, 28 and 42. Tissues were examined for arthritis and carditis, and *flaB* Q-PCR was performed on sub-inoculation site, heart base, ventricular muscle, tibiotarsus and quadriceps muscle. Inoculation sites of all mice were culture-positive at each interval tested, but none of the urinary bladders of mice inoculated with Δarp3 were culture-positive at 14 days, suggesting delayed dissemination, or reduced sensitivity due to lower tissue burdens (Table 2). Compared to inoculation site, urinary bladders were less consistently culture-positive in both groups of mice, underscoring the greater accuracy of PCR for assessing dissemination and tissue burdens (Table 3). At 14 days, 1/5 wild-type inoculated mice had 1+ inflammation of the tibiotarsus and 5/5 had carditis, whereas none of the Δarp3 inoculated mice had inflammatory lesions in joints or heart at this interval. At 28 days, 5/5 wild-type inoculated mice had both arthritis (1.6 ± 0.5 SD severity) and carditis, whereas only 1/5 Δarp3 inoculated mice had carditis and none had arthritis. At 42 days, 3/3 wild-type inoculated mice continued to have arthritis (1.5 ± 0.5 SD) and carditis, and 1/5 Δarp3 mice had arthritis (1+ severity) and 2/5 had carditis. PCR-positive tissue samples at all intervals indicated that wild-type infected mice had higher spirochete burdens in tissues compared to Δarp3 infected mice (Figure 2). At day 14, most tissues from wild-type inoculated mice were PCR-positive, whereas very few tissues from Δarp3 inoculated mice were PCR-positive (Table 3). The rate of PCR-positive tissues increased in the Δarp3 inoculated mice on days 28 and 42 to rates similar to wild-type infected mice, but *flaB* DNA copy numbers were consistently lower.

**Table 2:** **Outcome of infection of C3H mice with wild-type vs. *arp *null (Δarp3) *Borrelia burgdorferi *at intervals (days) after inoculation**

	**Culture**			**Inflammation**		
**Day**	**Inoculum**	**Inoc. site**	**Urinary bladder**	**Tibiotarsus**	**Knee**	**Heart**
14	wild-type	5/5*	3/5	1/5	0/5	5/5
	Δarp3	5/5	0/5	0/5	0/5	0/5
28	wild-type	5/5	4/5	5/5	4/5	5/5
	Δarp3	5/5	2/4**	0/5	0/5	1/5
42	wild-type	3/3	2/3	3/3	0/3	1/3
	Δarp3	4/4**	5/5	1/5	0/5	2/5

* number positive/number tested.

** one culture sample contaminated.

**Table 3:** **Rate of PCR (*flaB *DNA) positivity of sub-inoculation site, heart base, ventricular muscle, quadriceps muscle and tibiotarsus tissue from C3H mice at intervals (days) after inoculation with wild-type vs. *arp *null (Δarp3) *Borrelia burgdorferi***

**Day**	**Inoculum**	**SubIN**	**Hrt base**	**Vent M**	**Quad M**	**Tibiotarsus**
14	wild-type	4/5*	5/5	5/5	4/5	4/5
	Δarp3	2/5	1/5	0/5	0/5	0/5
28	wild-type	5/5	ND**	5/5	5/5	4/5
	Δarp3	4/5	ND	3/5	3/5	5/5
42	wild-type	3/3	3/3	3/3	3/3	3/3
	Δarp3	3/5	5/5	4/5	3/5	2/5

* number positive/number tested.

** ND = not done.

A confirmatory experiment was performed in which groups of 4 C3H mice were inoculated with 10^6^ wild-type or Δarp3 spirochetes, and then necropsied on day 28 to verify the difference in tissue spirochete burdens in heart base, ventricular muscle, quadriceps muscle, and tibiotarsal tissue. Tissues were not collected for histopathology. In wild-type infected mice, 4/4 inoculation sites and 3/4 urinary bladders were culture-positive, and 3/3 inoculation sites (one sample contaminated) and 0/4 urinary bladders were culture-positive in Δarp3 infected mice. Spirochete burdens were significantly lower (*P* ≤ 0.05) in tissues of Δarp3 infected mice compared to wild-type infected mice, including sub-inoculation site (139 ± 266 SD vs. 1,761 ± 1,682 SD), heart base (45 ± 54 SD vs. 2,333 ± 1,400 SD), ventricular muscle (28 ± 26 SD vs 448 ± 276 SD), and quadriceps muscle (15 ± 23 SD vs 367 + 291 SD). Spirochete burdens were also lower in tibiotarsus tissue of Δarp3 infected mice (13 ± 11 SD vs 16,171 ± 29,765 SD), but differences were not statistically different (*P* = 0.16). Based upon these observations, it was determined that both C3H-*scid* mice as well as C3H mice infected with Δarp3 had lower spirochete burdens in tissues.

Sera from C3H mice that were confirmed to be culture-positive at 60 days of infection with wild-type or Δarp3 spirochetes were determined to be appropriately sero-reactive against recombinant Arp antigen (Arp seropositive or seronegative, respectively). Serum antibody titers from Δarp3 infected mice were equivalent to antibody titers in mice infected with wild-type infected mice when tested against *B. burgdorferi* lysate antigen (≥1:24,300), and antibody titers to recombinant Arp antigen were verified to be either negative (Δarp3) or positive (Δarp3 + lp28-1G), with titers equivalent to Arp titers in wild-type immune sera (1:2,700).

Larval ticks were fed upon the before-mentioned wild-type or Δarp3 infected C3H mice 3 days before necropsy at day 42. Replete ticks were allowed to molt and harden into nymphs, and then tested by Q-PCR for *flaB* and *arp* DNA. Among ticks that fed upon wild-type infected mice, 30/30 were PCR positive for both *flaB* and *arp*, with 53,950 mean ± 84,668 SD *flaB* copy numbers per tick. In contrast, 40/50 ticks that fed upon Δarp3 infected mice were *flaB*-positive, with significantly lower spirochete loads per positive tick (1,384 mean ± 1,780 SD *flaB* copy numbers, *P* = 0.0002). Tick cohorts from individual Δarp3 infected mice contained 9/10, 5/10, 10/10, 6/10 and 10/10 positive ticks. Results demonstrated that Δarp3 can be acquired by ticks from infected C3H mice, but ticks that acquired Δarp3 harbored fewer organisms compared to wild-type.

**Figure 3:**
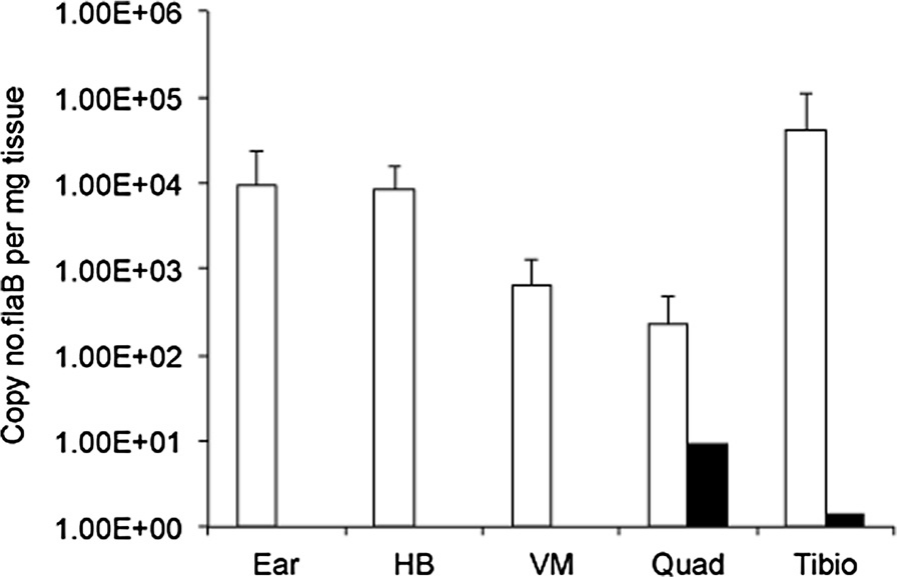
***Borrelia burgdorferi flaB *DNA copies per mg tissue weight (means ± standard deviations) in PCR-positive tissues, including ear, heart base (HB), ventricular muscle (VM), quadriceps muscle (QM) and tibiotarsus (Tt) of mice at 3 weeks after feeding of nymphal ticks from tick cohorts infected with wild-type or *arp *null Δarp3 *B. burgdoferi.***

The ability of Δarp3 spirochetes to be transmitted from infected ticks to naïve C3H mice was next evaluated by placing 10 nymphal ticks from the wild-type and Δarp3 positive tick cohorts (above) onto each recipient mouse. Mice were necropsied at 3 weeks following tick feeding, and ear, heart base, ventricular muscle, tibiotarsus and quadriceps muscle were tested by *flaB* Q-PCR. Among 5 mice fed upon by ticks carrying wild-type spirochetes, 4/5 mice became infected, and all tissue sites from the 4 positive mice were PCR-positive, with high copy numbers of *flaB* DNA in tissues (Figure 3). In contrast, 2 of the 7 mice that were fed upon by Δarp3 infected ticks were positive, but only a single tissue in each of the positive mice contained low copy numbers of *flaB* DNA. Results indicated that Δarp3 spirochetes are capable of tick-borne transmission. Since ticks infected with Δarp3 spirochetes had significantly fewer spirochete loads compared to ticks infected with wild-type spirochetes, it could not be concluded that there was less efficient transmission.

## Discussion

This study examined the effect of targeted deletion of BBF01/*arp* on infectivity of *B. burgdorferi* B31. The median infectious dose of *B. burgdorferi* B31 with an *arp* null mutation was elevated approximately ten-fold compared to wild-type spirochetes, and restored by complementation. Therefore, it is apparent that BBF01/*arp* is not essential for infectivity of the mammalian host. This is supported by indirect results of others, who demonstrated diminished infectivity in *B. burgdorferi* spirochetes lacking linear plasmid 28–1 (lp28-1), which encodes only two unique and functional genes, *vlsE* and *arp*[25-29]. Furthermore, clones of *B. burgdorferi* B31 with a deletion of the left side of lp28-1, which contains *arp*, remained infectious and capable of persistence, similar to wild-type spirochetes [25].

Examination of the pathogenicity of various *B. burgdorferi* B31 clones lacking lp28-1 has shown that clones lacking lp28-1 were infectious in BALB/c-*scid* mice and reached similar tissue burdens as wild-type spirochetes, but were incapable of inducing arthritis [29]. Results of the current study indicate that infection of C3H-*scid* mice with *arp* null spirochetes resulted in lower spirochete burdens in tissues with development of equally severe arthritis as that induced by wild-type spirochetes. Thus, it appears that *arp* null spirochetes are equally (if not more) arthritogenic than wild-type *B. burgdorferi* in C3H-*scid* mice. The lack of effect on tissue burdens and arthritis in BALB/c-*scid* mice infected with *B. burgdorferi* devoid of the entire lp28-1 plasmid, but reduced burdens in infections with *arp* null spirochetes observed in the current study are likely due to the experimental variations in *B. burgdorferi* strains (B31-5A11 vs. B31-A3), mouse strains (BALB/c-*scid* vs. C3H-*scid*), or a number of other possible genetic variables.

Lack of lp28-1 has been associated with failure to persist in immunocompetent mice. This has been attributed to *vlsE,* since clones lacking a region of the plasmid that encodes *arp* are capable of persistent infection [25,29]. The current study examined persistence in immunocompetent C3H mice up to 42 days after inoculation, and demonstrated that *arp* null spirochetes were indeed capable of persistence. In the present study, we also infected mice for antibody evaluation at 60 days of culture-confirmed infection, and thus verified persistence for up to 60 days. As in C3H-*scid* mice, *arp* null spirochete burdens were lower in C3H mouse tissues compared to wild-type spirochetes. Notably, arthritis severity was markedly reduced in C3H mice infected with *arp* null spirochetes. Since *arp* null spirochetes are fully arthritogenic in SCID mice, these results suggest that the lower pathogenicity of *arp* null spirochetes in immunocompetent mice is a consequence of susceptibility of the *arp* null mutant to immune response. Other antigens that are expressed during infection have also been shown to be susceptible to arthritis-resolving antibody responses, including DbpA [8], BmpA, and BmpB [30]. In the absence of Arp, these or other antigens may be targets of immune-mediated phenotypic effects noted in the present study.

Although *arp* null spirochetes are capable of surviving in the murine host, their ability to do so appears to be compromised, since *arp* null spirochete burdens were 2 logs fewer in tissues of SCID mice compared to wild type spirochetes, and were even lower in immunocompetent mice. Thus, *arp* null spirochetes appear to be either less fit to grow or are more vulnerable to innate and acquired immune factors compared to wild type spirochetes. This lack of fitness is likely responsible for the additional phenotypic effect of *arp* deletion that was observed in acquisition and transmission by vector ticks. Larval ticks were fed upon mice infected with wild-type or *arp* null spirochetes, and allowed to molt into nymphs. Ticks became infected with both types of spirochetes, but following molting, nymphal ticks that were colonized with *arp* null spirochetes had significantly lower spirochete loads per tick compared to ticks colonized with wild type spirochetes. The lower *arp* null spirochete loads were likely influenced by the lower spirochete burdens in tissues of mice that were fed upon by the ticks. This effect has been demonstrated by others [31] in which ticks that fed upon MyD88 deficient mice infected with *B. burgdorferi* had higher spirochete burdens compared to ticks that fed upon wild-type mice. MyD88 deficient mice have significantly higher spirochete tissue burdens compared to wild-type mice. The lower rate of transmission of *arp* null spirochetes from infected nymphal ticks to naïve mice could also have been influenced lower spirochete burdens in *arp* null colonized ticks. Further studies are needed to examine dynamics within ticks, but there is normally a significant burst of replication of spirochetes within fed ticks [32] that did not appear to occur in ticks colonized with *arp* null spirochetes. Nevertheless, results indicated that *arp* null spirochetes could be acquired and transmitted by vector ticks, albeit at diminished levels.

## Conclusion

Deletion of the *arp* gene resulted in a modest phenotypic effect, including reduced infectious dose, reduced fitness of *B. burgdorferi* for growth in the mammalian host, and reduced ability for acquisition and transmission by the vector tick. Deletion of a number of *B. burgdorferi* genes has been found to have only mild phenotypic effects upon infectivity and persistence of *B. burgdorferi* (reviewed in [33]). This is likely due in large part to compensatory up-regulation of other genes. Although the function of Arp remains unknown, the current study in which *arp* was deleted with relatively modest phenotypic effects underscores the complexity of *B. burgdorferi* biology and emphasizes caution in attributing phenotype or lack thereof to the role of a single gene alteration.

## Methods

### Mice

Specific-pathogen-free, 3 to 5 week old C3H/HeN (C3H) and severe combined immunodeficient (SCID) C3H/Smn.CIcrHsd-*Prkdc*^*scid*^ (C3H-*scid*) mice were obtained from Frederick Cancer Research Center (Frederick, MD) and Harlan Sprague Dawley, Inc. (Indianapolis, IN), respectively. Pregnant Swiss outbred Crl:CD1(ICR) mice were obtained from Charles River Laboratories (Hollister, CA). Mice were infected by subdermal inoculation of mid-log phase *B. burgdorferi* in 0.1 ml culture medium on the dorsal thoracic midline. Mice were killed by carbon dioxide narcosis and exsanguination by cardiocentesis. Infection status of mice was confirmed at necropsy by culture of the urinary bladder and sub-inoculation site, as described [4]. Animal use was approved by the University of California Davis Animal Care and Use Committee. University of California Davis has a Public Health Service Animal Welfare Assurance on file and is fully accredited by the Association for the Assessment and Accreditation of Laboratory Animal Care International.

### Histopathology

Joint (knee and tibiotarsus) and heart tissues were fixed in neutral buffered formalin, demineralized, paraffin-embedded, sectioned, and stained with hematoxylin and eosin. Tissues were blindly evaluated and tibiotarsal arthritis severity was scored on a scale of 0 (negative), 1 (mild), 2 (moderate) or 3 (severe). Based upon extensive use of this scoring system, a score of 3 is generally limited to SCID mice, and a score of 1–2 is typical of immunocompetent C3H mice [4,34,35]. The prevalence of carditis was also blindly recorded, but a severity score is not possible with carditis, due to variation in severity among mice within a particular treatment group, thereby precluding accurate scoring [34].

### Bacterial strains

Low passage infectious *B. burgdorferi* s.s. strain B31-A3 (wild-type) was acquired from D. Scott Samuels, University of Montana, and utilized as both a wild-type control and for genetic manipulation. B31-A3 is a clonal isolate of B31 MI, the prototype B31 strain utilized for genome sequencing [36,37]. An additional B31-A3 variant, *B. burgdorferi* B31-A3-lp28-1-G, containing a gentamicin resistance gene on lp28-1 [38], was provided by D. Scott Samuels (originally from P. Rosa, Rocky Mountain Laboratories). Spirochetes were grown in modified Barbour Stoenner Kelly (BSKII) medium [39] with 6% rabbit serum. Inocula were enumerated by dark-field microscopy using a Petroff-Hausser chamber immediately prior to use, and serial 10-fold dilutions were prepared for evaluating median infectious doses. For isolation of transformants, spirochetes were cultured on semi-solid gelatin-free BSKII medium supplemented with 1.7% dissolved agarose plus appropriate antibiotic (50 μg/ml streptomycin or 40 μg/ml gentamicin). *Escherichia coli* cloning strain TOP10F’ (Invitrogen, Inc., CA), was grown in Luria-Bertani broth under aerobic conditions at 37°C. Transformed *E. coli* were selectively cultured in broth medium with 50 μg/ml spectinomycin.

### Genetic modification of B. burgdorferi

Arp null mutants (Δarp) were constructed by exchange of the *arp* open reading frame (ORF) with a mutagenic cassette via homologous recombination. The mutagenic cassette consisted of a streptomycin-spectinomycin resistance cassette, *flaB*-*aadA* (kindly provided by D. Scott Samuels, University of Montana, Missoula, MT), flanked by regions of the *B. burgdorferi* B31-A3 plasmid lp28-1 that flanked the *arp* gene at both the 5′ and 3′ regions. Single Overlap Extension PCR (SOEing) was used to join each part of the mutagenic cassette through primers containing overlapping homology (Table 4). First, the 5′ flanking region (258bp) was amplified using primers ARP01 and the SOEing primer ARP02, which included homology to the 5′ region of the *flaB*-aadA PCR product. The *flaB*-*aadA* product (1199bp) was amplified using primers ARP03 and the SOEing primer ARP04, which included homology to the 5′ region of the 3′ region PCR product. The 3′ flanking region (1309bp) was amplified using primers ARP05 and ARP06. Each part was gel purified using the Qiagen Gel Extraction Kit (Qiagen Inc., Valencia, CA). SOEing was performed using a 2μl aliquot of each part mixed with 0.5 μl (10 μM) each of primers ARP01 and ARP06 to produce a fragment that was 2766bp in length. The resulting mutagenic cassette was cloned into the 3.9kb commercial vector, pCR2.1 TOPO (Invitrogen Corp., Carlsbad, CA) to produce a 7.5 kb suicide vector, “pKH-1”. Plasmid DNA of pKH-1 (5–10 μg) was electroporated into wild-type *B. burgdorferi* using the previously described protocol [40]. Transformants were selected by plating onto semi-solid BSKII medium (gelatin-free BSKII medium supplemented with 1.7% dissolved agarose and 50 μg/ml streptomycin). Clones that survived antibiotic selection were analyzed by PCR to confirm allele exchange using a combination of primers exterior and interior of the integration site (Table 4). PCR was performed to confirm the absence of the *arp* gene in several potential mutants. Plasmid profiling of Δarp mutants was performed by PCR as previously described [28] to select mutants that contained important plasmids, including cp9 (*rev*), cp26 (*ospC*), cp32-1 (BBP33), cp32-2/7 (BBO32), cp32-3 (*ospG*), cp32-6 (BBM32), cp32-8 (BBL32-34), cp32-9 (BBN32-33), lp17 (BBD12-13), lp21 (BBU06-07), lp25 (*pncA*), lp28-1 (*vlsE*), lp28-3 (BBH17), lp28-4 (non-coding region), lp36 (BBK12), lp38 (*ospD*), lp54 (*ospA*), and lp56 (BBQ67), using previously published primers [28,41]. One of the Δarp clones (Δarp3) that retained the same complete set of plasmids as the wild-type isolate was used in further experiments.

**Table 4:** **Primers for construction of the *arp *mutagenic cassette and verification of allelic exchange**

**Primer**	**Sequence (5′ > 3′)**	**Application**
ARP01	GCCTTTCGTTAAGGTTTTGTTT	amplify *arp* upstream homology
ARP02	GGAAATCTTCCTTGAAGCTCGGGTACAA	SOEing *arp* upstream homology
	GTTGTTCCTCCTAAATTAAATAAAAATAA	to *aadA* cassette
ARP03	TACCCGAGCTTCAAGGAAG	amplify *aadA* cassette
ARP04	GGTATATGTAATTTCGACTTTAAGTTAAAAAT	SOEing *arp* downstream
	CCGATTGTTTCATTTGCCGACTACCTTGGT	homology to *aadA* cassett
ARP05	GAACAATCGGATTTTTTAACTTAAAGTCG	amplify *arp* dowsteam homology
ARP06	ACCCCAGTAACTCAATTTCTAATTG	amplify *arp* dowsteam homology
ARP07	TTTCTTGATTAGGGTAAAAAATTCT	check integration at 5′ end
ARP08	GTCTTGTATTGTTGAACAAAACACTT	check integration at 3′ end
ARP09	GTTTCCATATGAGGGAAGCG	check integration within *aadA*
ARP10	CCAAGCGATCTTCTTCTTGTC	check integration within *aadA*

The Δarp3 clone was complemented with a whole lp28-1 plasmid that contained the *arp* gene and a selection marker for gentamicin (lp28-1-G). This plasmid was knocked in to replace the endogenous lp28-1 (where *arp* was deleted), as previously published [38]. Plasmid DNA containing lp28-1-G was purified from *B. burgdorferi* B31-A3-lp28-1-G, electroporated into B31-Δarp3 spirochetes, and then complemented transformants were selected with gentamicin. A series of PCRs using diagnostic primers (Table 1) were used to identify clones that had undergone successful plasmid exchange of lp28-1 *arp*::*aadA* with lp28-1G by confirming the presence of the *arp* operon. Plasmid profiling was performed and the complemented isolate B31-Δarp3-2.2 (Δarp3-lp28-1-G) was used for further analysis.

### Quantitative PCR

DNA was extracted from sub-inoculation site, heart base, ventricular muscle, quadriceps muscle and right tibiotarsus (integument removed), and samples were subjected to real-time Q-PCR. In addition to the site of inoculation, four additional sites were evaluated, based upon previous studies demonstrating that they all become consistently infected [22], but manifest different patterns of inflammation. Heart base is the site where carditis occurs, whereas cardiac ventricular muscle develops minimal or no inflammation [34]. In addition, the tibiotarsal joint typically develops arthritis, whereas the adjacent quadriceps femoris muscle develops minimal or no inflammation [42]. Quantification of gene copies was based upon copy number per mg of tissue weight, as previously described [22]. DNA was extracted from samples using the DNeasy tissue kit, according to the manufacturer’s instructions for tissues or insects (QIAGEN, Valencia, CA). In addition, DNA from *B. burgdorferi* cultured from mouse tissues was extracted for verification of genetic status of isolates. Three oligonucleotides, two primers and a probe, for the *B. burgdorferi flaB* and the *arp* genes were used, as previously described [19].

### Serology

Immune sera were generated in C3H mice inoculated with 10^5^ wild-type, Δarp3, or Δarp3 + lp28-1G spirochetes at 60 days of infection. Infection was verified by culture, and individual sera were tested by enzyme linked immunosorbent assay (ELISA) to verify the appropriate presence or absence of Arp-reactive antibody. Three-fold dilutions (starting at 1:300) of immune sera were titrated by ELISA for antibody to *B. burgdorferi* B31 lysates and recombinant Arp, as described [11]. Samples were tested in duplicate, and each assay included uninfected mouse serum as a negative control and wild-type infected mouse serum as a positive control.

### Tick acquisition and transmission

*Ixodes scapularis* ticks were acquired from Durland Fish, Yale University, as a single cohort of larvae from a pathogen-free laboratory-reared colony. In order to determine the ability of ticks to acquire infection, 40 larval ticks were placed on each mouse infected with either wild-type or Δarp3 spirochetes. Replete (fed) ticks were collected as cohorts from each mouse and allowed to harden and molt into nymphal ticks. Randomly selected ticks from each mouse/tick cohort were tested for *flaB* and *arp* by Q-PCR. Remaining nymphal ticks in each cohort were placed on naïve C3H mice to assess the relative ability of infected nymphal ticks to transmit wild-type or Δarp3 spirochetes.

### Statistical analysis

Multiple comparison analyses were performed using independent samples *t*-test or one-way analysis of variance, followed by post-hoc pair-wise comparisons (Tukey’s HSD test) (PASW Statistics v. 18.0). Calculated *P* values ≤ 0.05 were considered significant. The median infectious dose (ID_50_) was calculated using the method of Reed and Muench [43].

## Competing interests

The authors declared that they have no competing interests.

## Authors’ contributions

DI, KH, EH and SWB performed and analyzed results. SF, EH and SWB participated in experimental design. DI, KH, EH and SWB co-wrote the manuscript. All authors read and approved the manuscript.
